# Do Individuals in Old Age Prepare for the Risk of Long-Term Care? Results of A Population-Based Survey in Germany

**DOI:** 10.3390/ijerph15102189

**Published:** 2018-10-08

**Authors:** André Hajek, Thomas Lehnert, Annemarie Wegener, Steffi G. Riedel-Heller, Hans-Helmut König

**Affiliations:** 1 Department of Health Economics and Health Services Research, University Medical Center Hamburg-Eppendorf, 20246 Hamburg, Germany; infothomasl@gmail.com (T.L.); annemariewegener@web.de (A.W.); h.koenig@uke.de (H.-H.K.); 2 Institute of Social Medicine, Occupational Health and Public Health, University of Leipzig, 04103 Leipzig, Germany; Steffi.Riedel-Heller@medizin.uni-leipzig.de

**Keywords:** need for care, preferences, long-term care, old age, supplemental long-term care insurance, Germany

## Abstract

The aim of the present study was to identify specific actions and financial precautions undertaken by individuals in preparation for their long-term care needs, as well as to determine the correlates of these actions. A population-based survey of the German population aged 65 years and above (n = 1006) was used. Individuals were asked whether they have undertaken financial preparations for their long-term care needs (no; yes). With respect to specific actions, individuals were asked whether they (no; yes): (i) Had obtained information (e.g., from doctor, internet, care support center, care facility), (ii) had modified their home (e.g., installed a stair lift), and (iii) had moved (e.g., old-age housing, care in relatives’ homes). In total, 30.4% had undertaken financial preparations for their long-term care needs. With respect to the specific actions undertaken, 6.5% had obtained information, 4.8% modified their home, and 7.3% had moved. The outcome measure, ‘had modified home’, was positively associated with lower age, West Germany, and lower self-rated health. The outcome measure, ‘had moved’, was positively associated with being female, and higher education. The outcome measure, ‘financial preparations for long-term care needs‘, was positively associated with lower age, West Germany, higher education, being born in Germany, and private health insurance. It is alarming that only around one in three individuals aged 65 and older had undertaken financial preparations for long-term care needs, and that far fewer individuals had undertaken other actions to prepare for their long-term care needs. The provision of timely information regarding the risk of long-term care, as well as its associated costs, may assist in sustaining the satisfaction of long-term care recipients. It may also help to reduce the risk of long-term care for individuals in old age.

## 1. Introduction

It is well recognized that the number of individuals in old age will markedly increase in the next decades [1]. Due to the positive association between age and the need for long-term care, the number of individuals in need for long-term care will most likely rise considerably [2].

With regard to the German health care system, it is worth noting that health insurance is compulsory in Germany. In Germany, about 90% of the individuals are insured by statutory health insurance providers and the remaining 10% (mainly civil servants, self-employed individuals, and employed individuals above a certain income-threshold) are insured by private insurance. Access to the health care system is provided for all insured individuals. In 1995, mandatory long-term care insurance was introduced for the population in Germany. Long-term care insurance (statutory and private long-term care insurance) is administered by the health insurance system. Individuals in need of long-term care can choose between benefits in kind (professional care provided in private households, day and night care centers, as well as old age and nursing homes by public and private care providers) or cash benefits (e.g., if only informal care is used). However, the long-term care insurance is designed to cover many, but not all, long-term care services that may be required, resulting in substantial co-payments on the part of the care recipients.

As informal care is generally less costly than nursing home care (from the perspective of the social security system), health policy in Germany favors informal and formal caregiving in the community (home care) rather than nursing home care. It has recently been shown that around nine out of 10 older individuals in Germany preferred home care when long-term care was required [3]. Furthermore, individuals appreciated additional services (e.g., household assistance). Of those receiving nursing home care, more than 90% preferred a private room. Moreover, those residing in nursing homes were appreciative of the provision of a wide range of activities. In sum, it was concluded that (1) there might be a gap between individuals’ expectations of long-term care and the reality in Germany, and (2) programs to increase the awareness of private long-term care insurance may be important [3], as statutory long-term care insurance in Germany covers only around half of the formal nursing costs incurred. Need for long-term care is a major risk for individuals in old age [4,5]. However, most individuals delay dealing with this topic [6] and perceive themselves as badly informed [7]. In the same vein, it has been shown that only a small proportion of individuals have additional private long-term care insurance in Germany [8]. Previous studies have mostly focused on the question of whether individuals in old age have considered the topic of long-term care [7,9]. However, there is a lack of studies (i) identifying the actual precautionary efforts made by individuals in old age in preparation for their long-term care needs and (ii) examining the determinants of such efforts in Germany. Consequently, the purpose of this study was to identify the specific precautionary actions, including financial preparations, undertaken by individuals in preparation for long-term care needs, and to determine the correlates of these actions. This knowledge is of importance to care recipients, nursing services as well as policy makers. Specifically, this knowledge may assist in reducing the gap between long-term care preferences and reality [10]. This may help to increase the satisfaction of future care recipients, or reduce the likelihood of long-term care for individuals in later life.

## 2. Materials and Methods

### 2.1. Sample

Data for this study were drawn from a telephone survey (Computer Assisted Telephone Interview, CATI). To this end, n = 1006 individuals aged 65 and over were interviewed. A company, which specializes in market and social research, did the fieldwork in the year, 2015 (USUMA Berlin; Unabhängige Serviceeinrichtung für Umfragen, Methoden und Analysen; independent service for surveys, methods, and analyses in market and social research). A sample of registered private telephone numbers was randomly selected in order to achieve a representative sample. This was done in accordance with the Guidelines for Telephone Surveys (from the ADM Arbeitskreis Deutscher Markt- und Sozialforschungsinstitute e.V.). Additional computer-generated numbers also allowed for extra-directory households. Repeated calls were made (different times on different weekdays) until the call was answered. Out of the gross sample (n = 2346), n = 1006 interviews (42.9%) were conducted. Lack of time/interest (12.1%) and refusal to take part in telephone surveys (26.5%) were the two main reasons for non-participation. Further details have been published elsewhere [3,11,12,13,14]. Drawing on expert interviews [15] and a recently published literature review [16], a questionnaire was developed to assess long-term care preferences, as well as specific actions and financial preparations undertaken to prepare for long-term care needs. To this end, questions were developed based on existing questionnaires and were partially reformulated. Please see Hajek et al. [12] for further details.

Different pretests were undertaken to improve the questionnaire. This included evaluation conversations and a pilot study (n = 31). Moreover, the trained staff from USUMA received a glossary. In this glossary, the items and the underlying intentions were described in further detail.

Oral informed consent was given prior to assessment. Oral consent is common in survey research in Germany. The ethical guidelines of the International Code of Marketing and Social Research Practice by the International Chamber of Commerce and the European Society for Opinion and Marketing Research were followed.

### 2.2. Outcome Measures

With respect to financial preparations, individuals were asked whether (no; yes):

1. They had taken out additional long-term care insurance, and/or had prepared financially for their long-term care needs.

With respect to specific actions, individuals were asked whether (no; yes):

2. They had obtained information (e.g., from doctor, internet, care support centre, care facility);
3. They had modified their home (e.g., installation of a stair lift); and
4. They had moved (e.g., old-age housing, care in relatives’ homes).

### 2.3. Independent Variables

With respect to the independent variables, our regression model was adjusted for: Age, gender (men; women), educational level (without a vocational degree; apprenticeship, full-time vocational school; professional school or trade and technical school for vocational education; University, University of Applied Sciences, school of engineering), region (West Germany; East Germany), place of birth (Germany; born abroad), and having children (no; yes).

Moreover, additional independent variables were included as follows: Provided informal care for family/friends (no; yes), status of health insurance (statutory health insurance; private health insurance), need of care (level of care). To claim the benefits of the long-term care insurance in Germany, individuals must need a minimum of 90 minutes (per day) of assistance with the basic activities of daily living (e.g., using the toilet, eating, or bathing). Individuals are categorized into three care levels according to the level of care required. To this end, a nurse/physician of the medical service of the German statutory health insurance system assesses the abilities of the recipient. In our study, the level of care was dichotomized (0 = no level of care; 1 = level 1 to 3). This variable is comparable to variables explicitly measuring impairments in activities of daily living. Self-rated health was assessed on a five-point scale (from 1 = “very bad” to 5 = “very good”).

### 2.4. Statistical Analysis

Bivariate associations between independent variables and our four dichotomous outcome measures (1. Financial preparations; 2. Gathered information; 3. Structural measures; 4. Had moved) were calculated using *t*-test and chi-square procedures, as appropriate. In a second step, multiple logistic regressions were done to examine the correlates of our four outcome measures. The level of significance was set at *p* < 0.05. Statistical analyses were performed using Stata 15.1 (StataCorp, College Station, TX, USA).

## 3. Results

### 3.1. Bivariate Analysis

In our sample, the average age was 75.2 years in our study (SD: 6.6 years; 65 to 96 years). In total, 56.8% were female. About three out of four (74.6%) lived in West Germany. Bivariate associations are described in Table 1. In total, 30.4% had undertaken financial preparations for their long-term care needs. With respect to the specific actions undertaken, 6.5% had obtained information, 4.8% had modified their home, and 7.3% had moved.

Bivariate analysis revealed that ‘had obtained information’ was associated with higher age and higher education. The outcome measure, ‘had modified home’, was associated with the experience of providing care for family/friends. Moreover, the outcome measure, ‘had moved’, was associated with being female, and the experience of providing care for family/friends. In addition, the outcome measure, ‘financial preparations for long-term care needs’, was associated with lower age, being male, living in West Germany, higher education, being born in Germany, private health insurance, and better self-rated health. Further details are displayed in Table 1.

### 3.2. Regression Analysis

Results of multiple logistic regressions are displayed in Table 2. Regressions showed that ‘had obtained information’ was only positively associated with statutory health insurance [Odds ratio (OR): 0.38 (95%-CI: 0.15–0.98)]. The outcome measure, ‘had modified home’, was positively associated with lower age [OR: 0.94 (0.89–0.98)], West Germany [OR: 2.72 (1.04–7.10)], and lower self-rated health [OR: 0.71 (0.52–0.97)]. The outcome measure, ‘had moved’, was positively associated with being female [OR: 2.24 (1.25–3.99)] and higher education [university, polytechnic, school of engineering, OR: 3.98 (1.12–14.19)]. The outcome measure, ‘financial preparations for long-term care needs’, was positively associated with lower age [OR: 0.96 (0.93–0.98)], West Germany [OR: 2.67 (1.72–4.13)], higher education [professional school or trade and technical school for vocational education, OR: 2.45 (1.19–5.03)], being born in Germany [OR: 1.86 (1.06–3.27)], and private health insurance [OR: 2.98 (2.02–4.40)].

## 4. Discussion

The objective of this study was to identify the specific actions and financial preparations undertaken for long-term care needs and to determine the correlates of these actions, using a population-based survey of the German population ≥65 years. We found that only around one in three individuals had undertaken financial preparations for long-term care, and far fewer individuals had taken other specific actions.

The number of individuals who had undertaken financial preparations for long-term care needs are comparable to a nationally representative study (individuals aged 25 years; year 2014) in Germany (n = 1314). In this study, 23% of the individuals had supplementary long-term care insurance. In addition, individuals were asked about the actions they perceived could protect them against the financial risk of long-term care. Individuals perceived owning a house (61%) or saving money (66%) as protective actions [9]. They reported that these two measures may assist them should they require long-term care. However, it appears there are discrepancies between how individuals said they would act to prepare themselves against the financial risk of long-term care, and their realized actions. 

In another survey (n = 6218; insurees of a private health insurance aged 40 and above in Germany) [7], almost one out of two (48%) had reported that they had already considered the topic of long-term care in their own household. This number increases with age. Twenty-nine percent had considered their financial preparedness in terms of their long-term care needs, 22% had considered installing an emergency call system, and 21% had considered modifying their home. Only 4% of the individuals had considered the possibility of moving in with relatives/child to manage their long-term care needs, which is in accordance with our findings.

The positive association between ‘had obtained information’ and statutory health insurance could be explained by the fact that members of the statutory health insurance may be more concerned about a possible need for long-term care in Germany, and thus may be more proactive about the issue. However, this should be clarified in future studies.

There was a positive association between ‘had modified home’ and lower age, and lower self-rated health. The former link might be explained by the fact that younger individuals have better functional health, enabling them to make small structural modifications on their own (e.g., installing grab bars for bathrooms). The latter link appears plausible in that low self-rated health may represent an early sign of a (future) need for care, and this need for care could be dealt with by structural measures, such as a stair lift. Furthermore, the association between ‘had modified home’ and living in West Germany may be explained by differences in income and wealth. The association could also be explained by regional differences, due to historical reasons, in attitudes towards preparing for the future [17].

In our study, there was a positive association between ‘had moved’ and being female, as well as higher education. Both associations appear plausible to us because of the positive link between planning for the future and being female, and with education [18,19,20].

Moreover, undertaking financial preparations for long-term care needs was positively associated with (i) lower age, (ii) West Germany, (iii) higher education, (iv) being born in Germany, and (v) private health insurance. These positive links appear plausible because it has been shown that these groups also tend to have supplemental insurance, earn more, or have more disposable income.

In the same vein, a qualitative study (32 in-depth interviews and six focus groups) demonstrated that not purchasing long-term care insurance can be explained by perceptions that this form of insurance is “too costly”, and skepticism around the integrity and viability of private insurance companies, among other things. Family dynamics also play an important role, as does feeling adequately informed [21]. These findings have also been supported in quantitative studies [22].

Our study contributes to the scarce knowledge regarding the specific actions, including financial preparations, undertaken by individuals to prepare for their long-term care needs. The use of a population-based sample (65+) is a strength of this study, although the response rate was quite low and therefore the possibility of a sample selection bias cannot be ruled out. Due to the cross-sectional nature of this study, temporal relationships could not be observed. Another specific action that might be of importance for individuals in later life, but that were not included in this particular study, is the possibility of contracting agencies who place professional foreign caregivers. Future studies are needed to examine this in further detail.

## 5. Conclusions

The finding that only about one out of three individuals aged 65 and over had undertaken financial preparations for their long-term care needs is alarming, as is the finding that far fewer individuals had undertaken other specific actions to prepare for their long-term care needs. The provision of timely information regarding the risk of long-term care, as well as its associated costs, may assist in sustaining the satisfaction of long-term care recipients. It may also help to reduce the risk of long-term care for individuals in old age.

## Figures and Tables

**Table 1:** Bivariate associations between specific actions and financial provision in case of long-term care needs and independent variables.

	Had Gathered Information			Had Done Structural Measures			Had Moved			Financial Provision in Case of Long-Term Care Needs		
	No (*n* = 941; 93.54%)	Yes (*n* = 65; 6.46%)	*p*-Value	No (*n* = 958; 95.23%)	Yes (*n* = 48; 4.77%)	*p*-Value	No (*n* = 933; 92.74%)	Yes (*n* = 73; 7.26%)	*p*-Value	No (*n* = 689; 69.60%)	Yes (*n* = 301; 30.40%)	*p*-Value
**Age**: Mean (SD)	75.5 (6.5)	77.3 (7.5)	*p* < 0.05	75.7 (6.6)	74.5 (6.6)	*p* = 0.21	75.6 (6.5)	77.0 (7.2)	*p* = 0.07	76.3 (6.7)	74.2 (6.1)	*p* < 0.001
**Gender**: N (%)			*p* = 0.18			*p* = 0.34			*p* < 0.01			*p* < 0.01
Men	413 (94.72%)	23 (5.28%)		412 (94.50%)	24 (5.50%)		416 (95.41%)	20 (4.59%)		279 (64.58%)	153 (35.42%)	
Women	528 (92.63%)	42 (7.37%)		546 (95.79%)	24 (4.21%)		517 (90.70%)	53 (9.30%)		410 (73.48%)	148 (26.52%)	
**Region**: N (%)			*p* = 0.87			*p* = 0.08			*p* = 0.91			*p* < 0.01
East Germany	240 (93.75%)	16 (6.25%)		249 (97.27%)	7 (2.73%)		237 (92.58%)	19 (7.42%)		208 (81.89%)	46 (18.11%)	
West Germany	701 (93.47%)	49 (6.53%)		709 (94.53%)	41 (5.47%)		696 (92.80%)	54 (7.20%)		481 (65.35%)	255 (34.65%)	
**Education**: N (%)			*p* < 0.05			*p* = 0.37			*p* = 0.42			*p* < 0.01
Without a vocational degree	72 (96.00%)	3 (4.00%)		74 (98.67%)	1 (1.33%)		72 (96.00%)	3 (4.00%)		63 (85.14%)	11 (14.86%)	
Apprenticeship, full-time vocational school;	363 (95.53%)	17 (4.47%)		361 (95.00%)	19 (5.00%)		356 (93.68%)	24 (6.32%)		270 (72.58%)	102 (27.42%)	
Professional school or trade and technical school for vocational education;	220 (90.16%)	24 (9.84%)		229 (93.85%)	15 (6.15%)		223 (91.39%)	21 (8.61%)		167 (69.01%)	75 (30.99%)	
University, University of Applied Sciences; school of engineering	280 (93.02%)	21 (6.98%)		28 8 (95.68%)	13 (4.32%)		276 (91.69%)	25 (8.31%)		188 (62.88%)	111 (37.12%)	
**Place of birth**: N (%)			*p* = 0.35			*p* = 0.75			*p* = 0.26			*p* < 0.05
Born abroad	73 (96.05%)	3 (3.95%)		73 (96.05%)	3 (3.95%)		68 (89.47%)	8 (10.53%)		61 (80.26%)	15 (19.74%)	
Born in Germany	865 (93.31%)	62 (6.69%)		883 (95.25%)	44 (4.75%)		862 (92.99%)	65 (7.01%)		627 (68.67%)	286 (31.33%)	
**Having children**: N (%)			*p* = 0.13			*p* = 0.25			*p* = 0.19			*p* = 0.86
No	150 (90.91%)	15 (9.09%)		160 (96.97%)	5 (3.03%)		157 (95.15%)	8(4.85%)		113 (70.19%)	48 (29.81%)	
Yes	790 (94.05%)	50 (5.95%)		797 (94.88%)	43 (5.12%)		775 (92.26%)	65 (7.74%)		576 (69.48%)	253 (30.52%)	
**Status of health insurance**: N (%)			*p* = 0.11			*p* = 0.20			*p* = 0.85			*p* < 0.01
Statutory health insurance	797 (93.00%)	60 (7.00%)		819 (95.57%)	38 (4.43%)		794 (92.65%)	63 (7.35%)		629 (74.35%)	217 (25.65%)	
Private health insurance	140 (96.55%)	5 (3.45%)		135 (93.10%)	10 (6.90%)		135 (93.10%)	10 (6.90%)		59 (41.26%)	84 (58.74%)	
**Provided care for family/friends**: N (%)			*p* = 0.06			*p* < 0.05			*p* < 0.05			*p* = 0.32
No	459 (95.03%)	24 (4.97%)		468 (96.89%)	15 (3.11%)		456 (94.41%)	27 (5.59%)		339 (71.07%)	138 (28.93%)	
Yes	481 (92.15%)	41 (7.85%)		489 (93.68%)	33 (6.32%)		476 (91.19%)	46 (8.81%)		349 (68.16%)	163 (31.84%)	
**Level of care**: N (%)			*p* = 0.09			*p* = 0.18			*p* = 0.50			*p* = 0.07
No	53 (88.33%)	7 (11.67%)		55 (91.67%)	5 (8.33%)		57 (95.00%)	3 (5.00%)		45 (80.36%)	11 (19.64%)	
Yes	885 (93.85%)	58 (6.15%)		900 (95.44%)	43 (4.56%)		874 (92.68%)	69 (7.32%)		643 (69.07%)	288 (30.93%)	
**Self-rated health** (from 1 = ‘very bad’ to 5 = ‘very good’): Mean (SD)	3.6 (0.9)	3.6 (0.9)	*p* = 0.84	3.6 (0.9)	3.4 (0.9)	*p* = 0.06	3.6 (0.9)	3.6 (1.0)	*p* = 0.55	3.6 (0.9)	3.8 (0.9)	*p* < 0.01

Note: Comparisons between the two groups were done using *t*-test and chi-square procedures.

**Table 2:** Correlates of specific actions and financial provision in case of long-term care needs. Results of multiple logistic regressions (for each outcome measure: 0 = no; 1 = yes).

Independent Variables	Had Gathered Information (Ref.: No)	Had Done Structural Measures (Ref.: No)	Had Moved (Ref.: No)	Financial Provision in Case of Long-Term Care Needs (Ref.: No)
Age	1.03	0.94 **	1.03 ^+^	0.96 ***
	(0.99–1.07)	(0.89–0.98)	(0.99–1.07)	(0.93–0.98)
Sex (Ref.: Male)	1.25	0.64	2.24 **	0.81
	(0.70–2.23)	(0.35–1.16)	(1.25–3.99)	(0.60–1.11)
West and East Germany (Ref.: East Germany)	1.33	2.72 *	1.01	2.67 ***
	(0.67–2.66)	(1.04–7.10)	(0.54–1.88)	(1.72–4.13)
Apprenticeship, full-time vocational school (Ref.: Without a vocational degree)	1.12	5.11	1.97	1.79
	(0.35–3.63)	(0.44–59.55)	(0.57–6.73)	(0.89–3.60)
Professional school or trade and technical school for vocational education	2.71 ^+^	8.67 ^+^	3.14 ^+^	2.45 *
	(0.84–8.72)	(0.73–102.51)	(0.90–10.95)	(1.19–5.03)
University, University of Applied Sciences; school of engineering	2.36	5.16	3.98 *	1.90 ^+^
	(0.69–8.03)	(0.42–63.03)	(1.12–14.19)	(0.91–3.94)
German-born (Ref.: no)	2.54	1.61	0.67	1.86 *
	(0.66–9.83)	(0.50–5.17)	(0.30–1.51)	(1.06–3.27)
Children (Ref.: No children)	0.81	2.34 ^+^	1.37	1.45 ^+^
	(0.41–1.58)	(0.90–6.09)	(0.65–2.88)	(0.97–2.18)
Private health insurance (Ref.: statutory health insurance)	0.38 *	1.71	0.89	2.98 ***
	(0.15–0.98)	(0.85–3.45)	(0.43–1.85)	(2.02–4.40)
Provided care for family/friends (Ref.: no)	1.60 ^+^	2.10 *	1.50	1.21
	(0.92–2.77)	(1.16–3.80)	(0.89–2.53)	(0.90–1.62)
Level of care (Ref.: no)	0.42 ^+^	0.42 ^+^	1.90	1.48
	(0.17–1.01)	(0.15–1.14)	(0.52–6.96)	(0.71–3.08)
Self-rated health (from 1 = ‘very bad’ to 5 = ‘very good’)	1.06	0.71 *	0.98	1.07
	(0.78–1.44)	(0.52–0.97)	(0.73–1.31)	(0.91–1.27)
Constant	0.01 ^+^	2.05	0.00 ***	0.18
	(0.00–1.20)	(0.01–469.29)	(0.00–0.02)	(0.01–2.62)
Observations	994	994	994	981
Pseudo R²	0.053	0.088	0.044	0.096

Comments: Odd ratios were reported. 95% confidence intervals in parentheses. *** *p* < 0.001, ** *p* < 0.01, * *p* < 0.05, ^+^ *p* < 0.10. Ref. = Reference category.
